# Testing the Effectiveness of a Disclosure in Activating Children’s Advertising Literacy in the Context of Embedded Advertising in Vlogs

**DOI:** 10.3389/fpsyg.2020.00451

**Published:** 2020-03-17

**Authors:** Rhianne W. Hoek, Esther Rozendaal, Hein T. van Schie, Eva A. van Reijmersdal, Moniek Buijzen

**Affiliations:** ^1^Behavioural Science Institute, Radboud University, Nijmegen, Netherlands; ^2^Amsterdam School of Communication Research, University of Amsterdam, Amsterdam, Netherlands

**Keywords:** children, adolescents, YouTube, influencer marketing, advertising literacy, persuasion knowledge, disclosure, indirect measurement

## Abstract

Watching vlogs of social media influencers has become a favorite pastime for children and adolescents. For advertisers, vlogs are an excellent way to reach young viewers. As such, vlogs have become a powerful marketing tool. However, for children and adolescents it is often unclear whether a vlog contains advertising, which raises questions regarding the fairness and transparency of this type of advertising. If children do not recognize the commercial intent of in-vlog advertising, then they are unlikely to activate their advertising literacy, which may serve as a critical coping mechanism. The aim of this study was to investigate if a sponsorship disclosure stimulates children and adolescents’ (7–16 years old) to activate their advertising literacy when exposed to embedded advertising in vlogs and, subsequently, if advertising literacy activation is related to children’s brand attitude. Furthermore, we investigated whether the relation between exposure to a sponsorship disclosure and advertising literacy activation was moderated by children’s dispositional advertising literacy and their age. An innovative aspect of the current study is that advertising literacy activation was measured in two ways: with a self-reported questionnaire and via an indirect measurement task (Advertising Literacy Activation Task). The results showed that the children who were exposed to a sponsorship disclosure did not activate their advertising literacy to a higher extent than the children who were not exposed to such a disclosure. This might be because of the high prominence of the brand in the vlog; thus children may not have needed the disclosure to realize that the vlog was sponsored and accordingly activate their advertising literacy. The results also showed that stronger attitudinal advertising literacy activation led to a more negative brand attitude. Interestingly, this effect was only found when attitudinal advertising literacy was assessed with a questionnaire and not when it was assessed with the indirect measurement task. Thus, children who were more critical toward the in-vlog advertisement through self-reporting also had a more negative brand attitude. This suggests that direct and indirect measurements of advertising literacy activation reveal different processes through which children make sense of, and are affected by, advertising.

## Introduction

Watching online video’s is one of the favorite leisure time activities of today’s children and teens. They watch online video’s primarily on YouTube, but other platforms, such as Instagram, Snapchat, and the fast growing video-sharing platform TikTok, are used as well (OFCOM, 2018; Pew Research Center, 2018). One particular type of online video that is popular is the ‘vlog’. In this type of video blog, people share their daily life with viewers. Some vloggers, also referred to as ‘video content creators,’ have gained millions of followers (Lee and Watkins, 2016). For advertisers, vlogs are an excellent way to reach children; they are popular, widely viewed, and often provide a specific target group. Therefore, it is no surprise that in-vlog advertising is a powerful marketing tool (Lee and Watkins, 2016).

However, for children, the commercial intent of in-vlog advertising is often unclear because the commercial message is fully embedded in the entertaining content (Verdoodt, 2018). Embedded advertising, also referred to as product placement or sponsorship is a marketing technique where references to specific brands or products are incorporated into non-commercial media content with specific promotional intent (Van Reijmersdal et al., 2017). With embedded advertising, the boundaries between commercial and non-commercial online content (e.g., information or entertainment) are blurred (Wojdynski and Evans, 2016; Campbell and Evans, 2018). This embedded nature makes it hard for children to recognize the commercial intent of in-vlog advertising (Hudders et al., 2017), which raises questions regarding the fairness of this type of advertising. The main concern is that if children do not recognize the commercial intent of in-vlog advertising, they are unlikely to activate their advertising literacy (i.e., their general understanding of advertising’s persuasive intent and their skeptical attitude toward advertising). Activation of advertising literacy is important when processing in-vlog advertising, because it can help children to critically evaluate and cope with this type of advertising messages (Wright et al., 2005; Rozendaal et al., 2011b).

In order to help children better recognize in-vlog advertising and activate their advertising literacy while viewing it, vloggers are required to add a sponsorship disclosure to their video if it contains commercial content (e.g., FTC Advertisement Endorsements, n.d.). However, research on the effectiveness of sponsorship disclosures for in-vlog advertising among children is still scarce. More research has already been done into the effects of sponsorship disclosures in other embedded advertising formats, such as in-game advertising and product placement in television programs and movies among children (e.g., An and Stern, 2011; De Pauw et al., 2017) and adults (e.g., Boerman et al., 2012; Amazeen and Wojdynski, 2018; Campbell and Evans, 2018; Tessitore and Geuens, 2019). This research has shown that sponsorship disclosures can be an effective tool to help children and adults activate their dispositional advertising literacy (i.e., general knowledge of the commercial nature and critical attitudes toward advertising). Although these studies provide important insights into the effects of disclosures, it remains important to investigate the effectiveness of sponsorship disclosures in activating children’s advertising literacy in the context of in-vlog advertising, because children have less experience with this type of advertising. Moreover, vlogs can be very persuasive because many children adore their favorite vloggers and see them as important role models. As such, their processing of embedded advertising and the effectiveness of a disclosure might be different in this context.

The first aim of this study was to investigate if a disclosure can stimulate children’s advertising literacy activation when they are exposed to in-vlog advertising. Furthermore, we explored whether the relation between exposure to the disclosure and advertising literacy activation is moderated by children’s dispositional advertising literacy and their age. Our second aim was to investigate whether and how advertising literacy activation is related to children’s responses to the advertised brand (i.e., brand attitude). We focus on 7- to 16-year-olds because children in this age group are most interested in watching vlogs (Childwise, 2018) and because this age group encompasses two types of information processing: cued processing (common for children aged 7 to 11 years) and strategic processing (common for children aged 12 to 16 years; Roedder, 1981). The difference between cued and strategic processing is relevant because a disclosure can be seen as a cue that may be particularly useful for younger children in their processing of in-vlog advertising.

In this study, we adopt an innovative approach to measuring activation of children’s advertising literacy. Research on children’s advertising literacy activation traditionally measures this concept using self-reported questionnaires (e.g., Rozendaal et al., 2016a; De Pauw et al., 2017). The few studies that explored the effects of a sponsorship disclosure in sponsored vlogs (De Jans et al., 2019a; Van Reijmersdal et al., 2020) also used self-report questionnaires to measure the extent to which children activate their advertising literacy while watching sponsored vlogs. However, there are several disadvantages of using self-reporting (for an overview, see Hoek et al., 2019). The most important disadvantage is that questionnaires stimulate respondents to consciously and elaborately think about the processing of advertising. As a consequence, questionnaires may activate *post hoc* rationalizations (Vandeberg, 2014) that do not reflect the cognitive and affective processes that were actually activated during exposure to the advertising message. Therefore, in addition to measuring children’s advertising literacy activation via self-reporting, we also measure children’s advertising literacy activation using an innovative indirect measurement task (the Advertising Literacy Activation Task, Hoek et al., 2019).

### Children’s Advertising Literacy Activation and the Role of Disclosures

Advertising literacy, also referred to as persuasion knowledge (Friestad and Wright, 1994), includes a wide range of knowledge, attitudes, and skills needed to critically process advertising. Dispositional advertising literacy (i.e., knowledge and critical attitudes that are in the child’s mind regardless of exposure to advertising; Hudders et al., 2017) can be defined in at least two dimensions: conceptual and attitudinal advertising literacy. Dispositional *conceptual* advertising literacy includes, for instance, the understanding of advertising’s selling intent (John, 1999) and persuasive intent (Moses and Baldwin, 2005). Dispositional *attitudinal* advertising literacy includes a general disliking and skeptical attitude toward advertising (Rozendaal et al., 2011b). Dispositional advertising literacy gradually increases with age (e.g., John, 1999; Moses and Baldwin, 2005; Rozendaal et al., 2011b). It develops from simple to more sophisticated knowledge and beliefs about the nature and working of advertising (Wright et al., 2005; Rozendaal et al., 2011b; Hudders et al., 2017). The increase in children’s dispositional advertising literacy depends on both the development of children’s cognitive and social abilities, including information processing and perspective taking skills, and the socialization of children as consumers (John, 1999). That is, through experience in coping with advertising, children acquire advertising-related knowledge and attitudes that are relevant to their functioning as consumers (Moses and Baldwin, 2005).

To investigate if a disclosure can stimulate children to activate their dispositional advertising literacy when they are exposed to in-vlog advertising, the present study draws on insights from information-processing theory (Atkinson and Shiffrin, 1968) and schema theory (Anderson, 1978). Information-processing theory states that, rather than directly responding to incoming information, people first process this information and then respond accordingly (Atkinson and Shiffrin, 1968). Incoming information is first represented and stored in short-term memory, which only has limited capacity. In order for the information to be retained and stored for later use, it needs to be transferred to long-term-memory (Atkinson and Shiffrin, 1968; Roedder, 1981). New, incoming information can then serve as a cue to retrieve this stored information. Subsequently, the incoming information is processed in relation to the retrieved information that was already stored in long-term memory (Atkinson and Shiffrin, 1968; Roedder, 1981; Rozendaal et al., 2011b).

The information that is stored in long-term memory is believed to be organized in mental structures called schemas (Aronson et al., 2005). Schemas are knowledge structures that hold all information we have on a certain topic, for instance about advertising. Each schema consists of all relevant information associated with that topic and usually contains several subschemas (Anderson, 1978). In the case of the advertising schema, it is believed that there is one overarching schema that includes all general knowledge and beliefs about advertising (Dahlén and Edenius, 2007), with separate subschemas for dispositional conceptual and attitudinal advertising literacy (Friestad and Wright, 1994; Dahlén and Edenius, 2007; Evans and Park, 2015).

When combining the perspectives of information-processing and schema theories, they predict that exposure to advertising, which serves as a cue for information retrieval, should result in the activation of the advertising schema, including the subschema containing conceptual and attitudinal advertising literacy. As such, exposure to advertising could lead to the activation of children’s dispositional advertising literacy. However, information-processing theory also predicts that activating relevant information can be difficult for children. Even though information retrieval as a results of exposure to media content is largely automatic (Robinson and Neighbors, 2006), children also need to be able to use retrieval strategies (Roedder, 1981) in order to correctly use and link the retrieved information.

The activation of advertising literacy can be stimulated by exposing children to a ‘retrieval’ cue (Roedder, 1981; Hudders et al., 2017). Such a cue (i.e., incoming information) can result in the retrieval of related information (e.g., dispositional advertising literacy as stored in the advertising schema). A cue to stimulate children to activate their advertising literacy can, for instance, be a sponsorship disclosure, which is a forewarning about the persuasive intent of the message (Boerman et al., 2012; Rozendaal et al., 2016a). Earlier research has shown that disclosures can help children better recognize product placement as a type of advertising (De Jans et al., 2018). Furthermore, studies have shown that a visual disclosure can be helpful in activating children’s advertising literacy when it comes to embedded advertising in television programs and movies (e.g., De Pauw et al., 2017; Van Reijmersdal et al., 2017). For instance, a study by De Pauw et al. (2017) showed that children who were exposed to a visual warning cue (i.e., disclosure) before watching a movie clip with brand placement displayed higher levels of conceptual advertising literacy than children who were exposed to an auditory warning cue. Similar, a study by De Jans et al. (2019a) showed that including a disclosure in a vlog that contained advertising increased children’s attitudinal advertising literacy. In line with the results from previous studies, we set up our first hypothesis:

H1: Children who are exposed to a disclosure prior to in-vlog advertising will show higher levels of (a) conceptual and (b) attitudinal advertising literacy activation than children who are not exposed to a disclosure.

In order for children to activate their conceptual and attitudinal advertising literacy, these types of advertising literacy have to be present in the first place. When children do not have certain knowledge about the persuasive and selling intent of advertising in general (i.e., dispositional conceptual advertising literacy) or do not have a general skeptical attitude toward advertising (i.e., dispositional attitudinal advertising literacy), they will also not be able to activate this knowledge or attitude when they are exposed to advertising. Therefore, we argue that there will be an interaction effect between exposure to the disclosure and children’s dispositional advertising literacy. Specifically, we expect that the effect of the disclosure on advertising literacy activation will be stronger for children with higher levels of dispositional advertising literacy. This leads to the second hypothesis:

H2: The effect of the disclosure, as hypothesized in H1, will be stronger (weaker) for children with higher (lower) levels of (a) dispositional conceptual and (b) dispositional attitudinal advertising literacy.

### The Moderating Role of Age

Insights in children’s cognitive development suggest that activating relevant information from long-term memory is more difficult for younger children (i.e., younger than 12 years) than for older children (i.e., 12 years and older). As such, the processing of in-vlog advertising can differ markedly between younger and older children (Hudders et al., 2017).

The prefrontal brain plays a significant role in children’s ability to activate and retrieve relevant information from memory (Uytun, 2018) and doesn’t mature fully until late adolescence (Casey et al., 2005). However, from the age of 12 onwards, most children are able to use retrieval strategies to activate relevant information from memory and use it efficiently (i.e., strategic processors; Roedder, 1981; John, 1999), for instance, to use activated advertising literacy to counter advertising effects. Children younger than 12 years old are also able to do this, but only when they are prompted or cued (i.e., cued processors; Roedder, 1981; Rozendaal et al., 2011b). This implicates that, even though younger children may have a fairly high level of dispositional conceptual and attitudinal advertising literacy, activating this literacy and using it to decrease susceptibility may still be difficult for them (John, 1999). Children in this younger age group must be cued to activate their advertising literacy. Therefore, use of a disclosure is thus expected to be more important for activating advertising literacy in the younger age group than it is in the older age group. Thus, age was included as a moderating variable in this study. More specifically, we expect that age moderates the effect of the disclosure as hypothesized in H2. This leads to the following hypothesis:

H3: The interaction-effects, as hypothesized under H2, will be stronger for younger children (7–11 years old) than they will be for older children (12 years and older).

### Advertising Literacy Activation and Brand Responses

To investigate whether and how advertising literacy activation is related to children’s responses to the advertised brand (i.e., brand attitude), the present study draws on insights from the Persuasion Knowledge Model (PKM, Friestad and Wright, 1994) and reactance theory (Brehm and Brehm, 1981). The Persuasion Knowledge Model (Friestad and Wright, 1994) suggests that higher levels of advertising literacy activation can change the way children respond to an advertising message and its source (i.e., the brand). In the context of this study this means that when children become aware of the fact that a vlog is sponsored, they may realize that the vlog is not just entertaining or informative but is meant to persuade. This can trigger feelings about the honesty, trustworthiness, and credibility of the message. According to Brehm and Brehm (1981) reactance theory, people do not want to be manipulated and desire to maintain the freedom to feel and think what they want. Therefore, when advertising literacy schemas are active, people are assumed to use the activated knowledge and attitudes to cope with the persuasion attempt, which can result in negative responses (e.g., more negative brand attitudes).

Earlier research has found that, among adults, a better understanding of sponsored content resulted in a more critical attitude toward not only the sponsored content but also toward the brands (Boerman et al., 2014). However, the empirical evidence among children for this relationship is less conclusive. For example, with regard to *conceptual* advertising literacy, only few studies (De Jans et al., 2017) show that stronger activation of this concept leads to more negative brand responses. Interestingly, most studies report *positive effects* of conceptual advertising literacy activation on children’s brand responses (e.g., Rozendaal et al., 2009; Van Reijmersdal et al., 2015; Vanwesenbeeck et al., 2016, 2017; De Pauw et al., 2017). One explanation for the positive effect could be that children’s realization that the message is made to persuade (i.e., conceptual advertising literacy) actually persuades them to like, want, or buy the advertised product. In this study, we specifically focus on brand attitude as a brand response because it is an important indicator of how a brand is perceived and can predict people’s behavior toward the brand (Brown and Stayman, 1992). Based on the existing empirical evidence we hypothesize the following:

H4a: Stronger conceptual advertising literacy activation leads to a more positive brand attitude.

Earlier research on the relationship between *attitudinal* advertising literacy activation and children’s brand responses provides more convincing evidence for the defense view. These studies all show that children’s attitudinal advertising literacy has a negative effect on brand responses (e.g., Rozendaal et al., 2013, 2016a; Vanwesenbeeck et al., 2016, 2017). Thus, in contrast to conceptual advertising literacy, attitudinal advertising *does* seem to negatively influence brand responses, as suggested by the PKM (Friestad and Wright, 1994). An explanation for this could be that the negative attitude children have toward the specific advertising message, or toward advertising in general, spills over to the brand, leading to more negative brand responses. This is also referred to as affect transfer (Machleit and Wilson, 1988) and led to the following hypothesis:

H4b: Stronger attitudinal advertising literacy activation leads to a more negative brand attitude.

**FIGURE 1:**
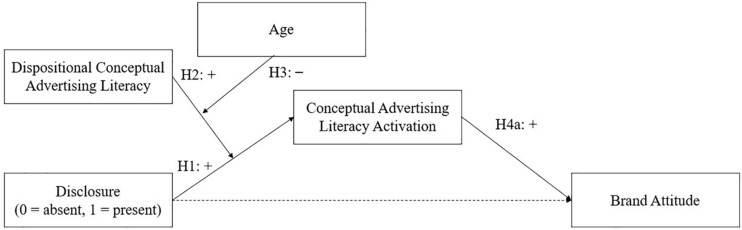
Conceptual model for hypotheses related to conceptual advertising literacy.

**FIGURE 2:**
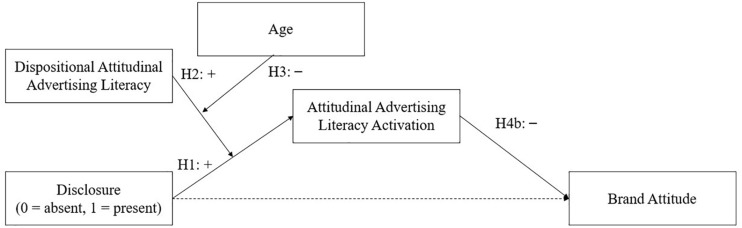
Conceptual model for hypotheses related to attitudinal advertising literacy.

The conceptual models for the hypotheses can be found in Figure 1 (conceptual advertising literacy) and Figure 2 (attitudinal advertising literacy).

### Measuring Children’s Advertising Literacy Activation

Over the last 10 years, several scholars have worked on the development of a valid and reliable measurement tool to assess children’s dispositional advertising literacy (e.g., D’Alessio et al., 2009; Ham et al., 2015; Rozendaal et al., 2016b). These tools aim to assess children’s advertising literacy that is present in the child’s intellect regardless of advertising exposure (i.e., knowledge and attitudes toward advertising in general; Hudders et al., 2017). In empirical studies in which children are exposed to specific advertisements to investigate their responses to those advertisements, these dispositional measures are often used in an adapted form to measure children’s advertising literacy in relation to those specific advertisements (i.e., situational advertising literacy; Hudders et al., 2017). For example, items such as “Is the purpose of advertising to sell products?” are adapted into “Is the purpose of *this* advertisement to sell products?” This way, researchers aim to measure whether children activate their general knowledge of advertising (i.e., dispositional advertising literacy) when they are confronted with a specific advertising message.

An issue with measuring children’s advertising literacy activation in this manner is that it is based on self-report questionnaires. Although questionnaires are suitable for assessing the dispositional dimensions of advertising literacy, they are considered less appropriate for assessing advertising literacy activation (Rozendaal et al., 2016b). There are several reasons for this. First, children have to retrieve information from memory in order to answer the questions in the questionnaire. This can be difficult for children, because this means that they have to introspectively search for the answers (Dunham et al., 2006). With respect to advertising literacy activation, retrieving information from memory is especially hard, because the questions would relate to the processing of advertising, which is usually automatic and subconscious (Vandeberg et al., 2013). Second, the wording of the questions and answering options may prompt the answers that children give (Brace, 2004). In this case it could mean that children choose the option that is most appealing to them, or that is in line with what they think the researcher wants to know. Third, and most importantly, questionnaires stimulate respondents to consciously and elaborately think about the processing of advertising. As a consequence, questionnaires may activate *post hoc* rationalizations (Vandeberg, 2014) that do not reflect the cognitive and affective processes that were actually activated during exposure to the advertising message. Concluding, the self-report measurement tools that are currently used to assess children’s dispositional advertising literacy have several disadvantages that make them less suitable to assess children’s advertising literacy activation.

An alternative way in which children’s activation of advertising literacy can be measured, and a way with which the disadvantages of self-report can be avoided, is the use of indirect measures (e.g., reaction time measures). The use of indirect measures is very common in schema activation research (e.g., Kim and Hancock, 2016; Leavitt et al., 2016). One of the most important advantages of indirect measures is that they do not require people to consciously reflect on a past experience (Vandeberg et al., 2013). Therefore, they are better able to capture subconscious processes (De Houwer, 2006), such as the activation of dispositional advertising literacy as a response to advertising exposure. Although the use of indirect measures with children is not yet very widespread, previous studies have shown that it is possible to use these types of reaction time measures when doing research with children aged 6 years and older (e.g., Baron and Banaji, 2006; Banse et al., 2010; Cvencek et al., 2011).

In the current study, in addition to a self-reported measure, a new indirect measurement task was used to assess children’s advertising literacy activation: The Advertising Literacy Activation Task (ALAT; Hoek et al., 2019). The ALAT is based on the idea that when a certain concept (i.e., schema) is activated, it is easier to process and categorize words related to this concept (i.e., schema). A concept is usually active in relation to a real-life situation or condition (e.g., being in a supermarket activates thoughts about supermarkets; Robinson and Neighbors, 2006), but can also be activated by exposure to media content (Robinson and Neighbors, 2006). For example, exposure to an advertising message can activate thoughts about advertising (i.e., the advertising schema, including dispositional conceptual and attitudinal advertising literacy). The extent to which a schema is activated can be derived from the speed and accuracy with which a person can place related and unrelated words into a relevant category (Fazio and Olson, 2003; Robinson and Neighbors, 2006). This has been shown in several categorization tasks within priming research (e.g., Fazio et al., 1986; Zeelenberg et al., 2003; Ortells et al., 2016). For instance, Fazio et al. (1986) showed that people are faster in categorizing positive words when they were presented with a positive prime prior to categorizing the positive word.

In the ALAT, the categorization task is designed in such a way that children are asked to categorize targets, in this case advertising-related words (e.g., buy), non-advertising related words (e.g., weather) and attitudinal words (e.g., boring) into one of two categories: advertising or news. The ALAT is an indirect measurement task, because it does not directly ask participants questions regarding their advertising literacy activation, but indirectly assesses it from their reactions on the task (Fazio and Olson, 2003). The two most important premises of the ALAT are that (1) exposure to an advertising cue (e.g., an advertisement and/or sponsorship disclosure) activates dispositional conceptual and attitudinal advertising literacy, which (2) in turn increases accuracy and speed of categorizing words associated with advertising.

To explore if the way in which advertising literacy activation is measured (i.e., indirect measurement vs. self-report) affects the hypothesized relationships as depicted in Figures 1, 2, we formulated the following research question:

RQ: To what extent do the hypothesized relationships between the sponsorship disclosure, children’s level of advertising literacy activation and their responses to the advertised brand differ when advertising literacy activation is measured via an indirect measurement task compared to a self-report questionnaire?

## Materials and Methods

We conducted an experiment with a one-factor (disclosure vs. no disclosure) design. The experiment was conducted over 2 weeks in July 2018 in a science museum for children in Amsterdam, the Netherlands. The study was approved by the Ethics Committee of the Social Sciences Department at Radboud University, the Netherlands (ECSW2017-1303-492).

### Participants

In total, 289 participants took part in the experiment. Thirty-one participants were excluded from the data analysis for one of the following reasons: (1) They did not sufficiently master the Dutch language, (2) The manipulation failed (e.g., the participant saw both versions of the stimulus material), (3) They did not finish the entire experiment, (4) They did not fall within the age range for this study (i.e., younger than 7 years old or older than 16 years old), or (5) Their performance on the indirect measurement task was not good enough (i.e., more than 25% of their data was removed after outlier analysis). Of the participants that remained (*N* = 258), 50.0% were girls. The participants’ mean age was 10.45 (*SD* = 1.94, range: 7–16). Almost two-thirds of the participants (65.5%) were in primary school, and 34.5% of the participants were in secondary school.

### Procedure

Data collection took place in the science museum, which has a designated area where scholars are allowed to do research. This research area functioned as a research lab where the experiment was conducted under controlled conditions. The research area consists of two rooms: one room where participants and their parents were given details about the study and parents signed the consent form and one room where the children actually participated in the study. In the second room, four desks were each equipped with a laptop, a headphone, a mouse, and a button box. Recruitment of the participants took place inside the museum. Participants were told that the study was about vlogs and the way that people process information presented in vlogs. When they (and their parents) agreed to participate, they were taken to the area where the study took place. The consent form was signed by a parent or guardian for all children. After signing the consent form, participants were taken to the room where the actual study was conducted. They were positioned behind one of the laptops and asked to put on the headphone.

In the first part of the study, participants watched a vlog that contained either a disclosure at the beginning of the vlog (experimental condition) or not (control condition). Next, they performed the indirect measurement task to assess their advertising literacy activation. Finally they filled out a questionnaire. The experiment took approximately 20 min to complete. After completion, participants were thanked for their contribution and given a debriefing letter which explained the purpose of the study. None of the participants received an incentive for participating in the study.

### Stimulus Material

The vlog used in this study was selected on the basis of three selection criteria. First, the vlog needed to be attractive for children of all ages (e.g., we did not want to use a vlog that would be perceived as being childish). Second, the product/brand in the vlog needed to be attractive for children of all ages. Third, the product was preferably easy to buy for the participants (e.g., not too expensive and easily available). Based on these criteria, we selected a video of a Dutch male vlogger, who has approximately 146,000 followers on YouTube. In the vlog, he visits a popular pizza chain to make pizzas for a (sponsored) pizza lunch at a secondary school. He then visits the school where the children have to complete a set of challenges in order to win the lunch. The original vlog lasted 7 min and 3 s. For the purpose of this study some parts of the vlog were removed (e.g., a fragment in which the vlogger drives to the school, which was not necessary for the narrative). The final version of the vlog used in this study lasted 4 min and 36 s.

The selected vlog includes several features which are typical for (sponsored) vlogs. First, the vlogger films himself using two main camera angles: one where the camera is focused on the environment (e.g., the vloggers’ house or a shopping street) and another that’s selfie-style. Second, the vlogger films himself in ordinary settings (e.g., on the street, in a pizza store, in a class-room) while constantly providing engaging, improvised commentary as he films. Third, the vlog shows a give-away, which is an persuasive tactic that is often used in sponsored vlogs. The give-away consists of winning a pizza lunch from the sponsoring pizza chain for the entire class.

From one of our earlier studies (Van Berlo et al., 2017), in which we extensively pretested stimulus materials including brand placement for a similar age group, we know that ‘pizza’ is a highly relevant product category for children and teenagers. With regard to the selected pizza chain brand, this study showed that most children were familiar with the brand and held neutral attitudes toward it (important to prevent a ceiling effect on brand attitude).

For the experimental condition, we added a disclosure at the start of the video because a disclosure at the beginning of a video is more effective than a disclosure concurrent with the product placement (De Pauw et al., 2017). The disclosure was portrayed in white letters on a black screen, reading *“[name vlogger] has been paid by [name company] to advertise in this video”* (Van Reijmersdal et al., 2020). The disclosure was based on social media advertising codes (FTC, 2013; WOMMA, 2013) and was shown for 10 s. In the control condition, we added a black screen without any message at the beginning of the video. Like the disclosure message (which was portrayed in white letters on a black screen), the black screen was shown for 10 s. In doing so, we kept the length of the two videos and the time between the start of the video (i.e., the moment when the participant clicks the start button) and the actual start of the vlog content (i.e., when the vlogger comes into the picture) exactly the same. Thus, participants in both conditions saw a black screen during the first 10 seconds of the video. The only difference was that in the experimental condition, the disclosure text was added to the black screen.

### Measurements

#### Children’s Advertising Literacy Activation

Children’s advertising literacy activation was assessed with both an indirect measurement task (the ALAT) and a (direct) self-reported questionnaire measure.

##### Indirect measurement task

Directly after watching the video, children performed the indirect measurement task: The Advertising Literacy Activation Task (ALAT) as described in Hoek et al. (2019). In the ALAT, participants are asked to categorize words. These words are either related to advertising (congruent with stimulus material, e.g., buy, product) or news (incongruent with stimulus material, e.g., anchor, information). Furthermore, attitudinal words (e.g., boring, irritating) are included in the task. For a complete list of the words used, see Table 1. Each word had to be categorized twice. Both the accuracy and speed of the categorization were recorded. Accuracy was registered as either 0 (incorrect response) or 1 (correct response). The speed of the categorization was recorded in seconds with millisecond accuracy. The ALAT was made and executed in PsychoPy version 1.84.2. We used a button box instead of a regular keyboard in order to get reaction times with millisecond accuracy.

**TABLE 1:** Words used in the Advertising Literacy Activation Task.

Practice	Advertising	News	Attitudinal
Order	Product	Jeugdjournaal^1^	Annoying
Purchase	Advertisement	NOS	Boring
Brand	Discount	Journaal^2^	Fun
Pay	Price	Reporter	Interesting
Journalist	Buy	Event	Funny
Studio	Persuade	Weather	Stupid
Domestic	Offer	Informative	Fake
Foreign	Stuff	Countries	Mislead
	Tempt	Information	Lies
	Store	Anchor	Lying

*^1^Jeugdjournaal is the name of a news broadcast especially for children in country of this study. ^2^Journaal is the name of a regular news broadcast in the country of this study.*

Both accuracy and speed of categorizing the advertising-related words were considered as indicators for advertising literacy activation. More accurate and faster categorization of the advertising-related words shows a higher level of advertising literacy activation (Fazio and Olson, 2003). We looked separately at the conceptual advertising words (as indication for conceptual advertising literacy activation) and the attitudinal advertising words (as indication for attitudinal advertising literacy activation). Note that for the attitudinal words, only *negative* words were considered to be advertising-related because dispositional attitudinal advertising literacy is the extent to which one has a negative and skeptical attitude toward advertising (Rozendaal et al., 2011b).

In the final dataset, seconds were converted to milliseconds. Outliers in response time were removed via the method described by Leys et al. (2013), where the absolute deviation around the median is used to calculate outliers. We constructed four variables that indicated Advertising Literacy Activation. These four variables were all based on the calculation of a difference score between the congruent (advertising and negatively valenced) words and the incongruent (news and positively valenced) words. A difference score is needed to account for individual differences in response speed or speed accuracy trade-off that are unrelated to this specific task.

We created four difference scores: two for conceptual advertising literacy activation and two for attitudinal advertising literacy activation. The difference score for categorization of the conceptual words (i.e., conceptual advertising literacy activation) was calculated by subtracting the value on the categorization of the news words (incongruent words) from the value on the categorization of the advertising words (congruent words). A higher score would then indicate stronger advertising literacy activation, because a higher score means that the child accurately categorized more advertising words than news words. The difference score for speed of categorization of the conceptual words (i.e., conceptual advertising literacy activation) was calculated by subtracting the mean reaction time value on the advertising words (congruent words) from the mean reaction time value on the news words (incongruent words). This way, a higher score again indicated stronger advertising literacy activation, because a higher score means that the child was faster in categorizing the advertising words than in categorizing the news words. The same was done for the attitudinal words, where the difference for categorization and speed of categorization were calculated in the same way, but with the negative words as congruent words and positive words as incongruent words.

##### Self-report questionnaire measurement

The second measure of children’s advertising literacy activation was a self-reported questionnaire (part of the general questionnaire, see below). Children’s conceptual advertising literacy activation was measured with six questions. Two were related to advertising recognition (‘Did this video contain advertising?’ and ‘Was this video sponsored by a brand?’). Four were related to the commercial intent (‘Is the aim of this video to ….’ followed by “make people like brand X,” “make people want to have brand X,” “make people think positively about brand X,” and “make people buy brand X”; Van Reijmersdal et al., 2020). The scale ranged from 1 (*no, definitely not*) to 6 (*yes, definitely*). Factor analysis showed one dimension (*EV* = 3.32, *R*^2^ = 0.55). Mean scores were calculated (Cronbach’s alpha = 0.84, *M* = 4.40, *SD* = 1.09, range 1 to 6). Children’s attitudinal advertising literacy activation was measured with four questions (‘What do you think about the presence of brand X in the video? Do you think that is ….’ followed by “honest” (R), “bad,” “good” (R), “wrong”; Van Reijmersdal et al., 2020). The scale anchors were adjusted to the questions. For example 1 (*totally not honest*) to 6 (*very honest*). Factor analysis showed one dimension (*EV* = 2.43, *R*^2^ = 0.61). Mean scores were calculated (Cronbach’s alpha = 0.78, *M* = 2.54, *SD* = 0.91, range 1 to 6).

#### Dispositional Advertising Literacy

Children’s dispositional conceptual advertising literacy was assessed with five questions (‘Is the aim of advertising to ….’ followed by “make you want to buy the advertised product,” “make you want to have the advertised product,” “make you think positively about the advertised product,” “to make you feel positively about the advertised product,” “seduce you to buy the advertised product”; Rozendaal et al., 2016b). The scale ranged from 1 (*no, definitely not*) to 6 (*yes, definitely*). Factor analysis showed one dimension (*EV* = 2.86, *R*^2^ = 0.57). Mean scores were calculated (Cronbach’s alpha = 0.81, *M* = 5.13, *SD* = 0.93, range 1.60 to 6). Dispositional attitudinal advertising literacy was measured with 10 questions (‘How often do you think advertising is ….’ followed by “fun” (R), “misleading,” “funny” (R), “boring,” “stupid,” “annoying,” “honest” (R), “truthful” (R), “believable” (R), “fake”; Rozendaal et al., 2016b). The scale ranged from 1 (*never*) to 6 (*always*). Factor analysis showed two dimensions: *EV* = 4.10, *R*^2^ = 0.32 for items related to disliking and *EV* = 1.86, *R*^2^ = 0.28 for items related to skepticism. However, the two dimensions were considered together to reduce the number of analyses needed to test the hypotheses. The reliability level for the nine items is good (excluding ‘misleading,’ Cronbach’s alpha = 0.83, *M* = 4.19, *SD* = 0.80, range 1.60 to 6).

#### Brand Attitude

Brand attitude was measured with six questions [‘Do you think brand X is ….’ followed by “nice,” “nasty” (R), “good,” “stupid” (R), “tasty,” “bad” (R); Van Reijmersdal et al., 2020]. The scale ranged from 1 (*totally not nice*) to 6 (*very nice*). Factor analysis showed one dimension (*EV* = 4.00, *R*^2^ = 0.67). Mean scores were calculated (Cronbach’s alpha = 0.90, *M* = 4.78, *SD* = 0.93, range 1 to 6).

#### Control Variables

As control variables, we assessed sex, type of school (primary/high school), and grade. In addition, we measured brand recall and brand recognition. Brand recall was measured with one question (‘Which brand or brands did you see in the video?,’ 49.6% correct) followed by one question assessing brand recognition (‘Which of the following brands did you see? This can be one brand, several brands, or no brand.’ Participants were presented a list of six brands including three pizza brands, 89.9% correctly identified the brand). We also measured prior exposure to the specific video (10.5% yes), familiarity with the vlogger (61.6% yes), and brand familiarity (93.4% yes). We also measured how often children watched videos from this vlogger (*M* = 1.69, *SD* = 0.88, range 1 [*never*] to 6 [*every day*]) and brand use (*M* = 1.94, *SD* = 0.85, range 1 [*never*] to 6 [*every day*]). We also assessed the responses to the video and vlogger (based on Van Reijmersdal et al., 2020). Attitude toward the video and the vlogger were both measured with one question (‘Please give X [the video]/[name vlogger] a grade’) on a scale ranging from 1 (*most negative*) to 10 (*most positive*). Both the video and the vlogger were rated positively (*M*_*Video*_ = 7.26, *SD*_*Video*_ = 1.61, range 1 to 10, *M*_*Vlogger*_ = 7.31, *SD*_*Vlogger*_ = 1.75, range 1 to 10). All of the variables were measured with one question each.

### Manipulation Check

We included two questions to check if participants perceived the manipulation as intended. First, we asked them if they saw a text at the beginning of the video (answer options: yes; no; I don’t know). A Chi^2^ analysis showed that the manipulation was successful. In the condition with the disclosure, 78.0% of the children reported seeing a disclosure versus 14.3% of the control condition, χ^2^(2, *N* = 258) = 107.78, *p* < 0.001. We then asked them which text they saw. There were five answer options. One answer option was correct (“[name vlogger] is being paid by [name company] to advertise in this video”). Three answer options were incorrect (1) “This is a video from YouTube,” (2) “[name vlogger] hopes that you like this video, click on the thumps up,” and (3) “YouTube earns money by airing this video”). There was also one option mentioning not seeing the text. Of the children in the disclosure condition who indicated that they saw a disclosure, 70.9% identified the correct disclosure text. Of the children in the control condition, 6.2% indicated to have seen the disclosure text that was shown in de experimental condition (which they did not see). Most participants in the control condition indicated correctly that they had not seen any text (68,2%).

### Plan of Analyses

All analyses were done in SPSS (Version 25). We first performed several randomization checks to see which variables needed to be included as covariates in the main analyses. Randomization checks were all χ^2^ and *t*-test analyses. Second, we tested the hypotheses by doing two moderated mediation analyses. Both analyses were done with brand attitude as the outcome variable and condition (i.e., exposure to the disclosure) as the predictor variable. The first analysis tested the model presented in Figure 1 with dispositional conceptual advertising literacy as the first moderator variable. Age was included as a second moderator. For this, age was recoded into two categories: 7- to 11-year-olds (i.e., cued processors Roedder, 1981; *n* = 181; *M* = 9.45, *SD* = 1.21; 52% were girls) and 12- to 16-year-olds (i.e., strategic processors; Roedder, 1981; *n* = 77; *M* = 12.82, *SD* = 1.10; 46% were girls). This analysis had three mediator variables. Two of the mediators were assessed with the indirect measurement task and meant to indicate conceptual advertising literacy activation (difference scores for accuracy of categorizing the conceptual words and speed of categorizing the conceptual words). We further included conceptual advertising literacy activation as assessed by the self-reported questionnaire as a mediator. The three mediators were tested in parallel.

The second analysis tested the model presented in Figure 2 with dispositional attitudinal advertising literacy as the first moderator variable and age group as the second moderator. This analysis also had three mediator variables. Two of the mediators were assessed with the indirect measurement task and meant to indicate attitudinal advertising literacy activation (difference scores for accuracy of categorizing the attitudinal words and speed of categorizing the attitudinal words). We further included attitudinal advertising literacy activation as assessed by the self-reported questionnaire as a mediator. The three mediators were tested in parallel. Both models require moderated mediation analysis—we used Hayes (2017) SPSS PROCESS macro version 3.2.01, model 11 to test the hypotheses simultaneously. The default bootstrapping procedure was applied with 95% bias corrected accelerated confidence intervals based on 10,000 bootstrap samples.

## Results

Table 2 shows all means, standard deviations, and zero-order correlations for the variables of interest.

**TABLE 2:** Means, standard deviations, and Pearson correlations for all variables related to advertising literacy and brand attitude.

	*M*	*SD*	2	3	4	5	6	7	8	9	10
(1) Child age	10.45	1.94	0.36***	0.06	0.52***	–0.02	0.23***	–0.05	−0.13*	0.06	0.20**
(2) Dispositional conceptual advertising literacy	5.13	0.93		0.08	0.49***	0.05	0.18**	0.15*	–0.06	0.13	0.17**
(3) Dispositional attitudinal advertising literacy	4.19	0.80			0.05	0.31***	–0.07	0.27***	–0.09	0.13	−0.18**
(4) Conceptual advertising literacy activation (self-report)	4.40	1.09				0.10	0.12	0.03	–0.08	0.07	0.09
(5) Attitudinal advertising literacy activation (self-report)	2.54	0.91					0.06	0.11	–0.03	0.02	−0.46***
(6) Categorization of conceptual words (DS)	–0.08	0.22						0.18**	0.20***	0.02	0.13*
(7) Categorization of attitudinal words (DS)	0.67	0.73							–0.08	0.27***	0.00
(8) Speed of categorizing conceptual words (DS)	–54.44	189.20								−0.28***	0.02
(9) Speed of categorizing attitudinal words (DS)	194.15	601.21									–0.03
(10) Brand attitude	4.78	0.93									

DS = Difference Score, difference score is calculated in such a way that a higher score means stronger advertising literacy activation. *** *p* < 0.001, ** *p* < 0.01, * *p* < 0.05.

### Randomization Check

We ran a number of randomization checks to see which variables needed to be included as covariates in the main analyses. The following variables were equally distributed across both conditions: sex, χ^2^(1, *N* = 258) = 0.25, *p* = 0.618; brand recall, χ^2^(1, *N* = 258) = 0.76, *p* = 0.385; brand recognition, χ^2^(1, *N* = 258) = 0.91, *p* = 0.341; having seen the video before, χ^2^(2, *N* = 258) = 5.25, *p* = 0.073; familiarity with the vlogger, χ^2^(2, *N* = 258) = 0.03, *p* = 0.984; video rating, *t*(256) = 0.76, *p* = 0.448, 95% CI [−0.24; 0.55], *d* = 0.01; vlogger rating, *t*(256) = 1.42, *p* = 0.157, 95% CI [−0.12; 0.74], *d* = 0.02; brand familiarity, *t*(256) = 0.41, *p* = 0.680, 95% CI [−0.23; 0.35], *d* = 0.01; and brand use, *t*(256) = 0.56, *p* = 0.579, 95% CI [−0.15; 0.27], *d* = 0.01. These variables were therefore not included as covariates in the main analyses. For the variable watching vlogs of this vlogger, we found a difference between the two conditions. In the experimental condition, children indicated that they watch vlogs of this vlogger less often (*M* = 1.55, *SD* = 0.80) than children in the control condition (*M* = 1.85, *SD* = 0.94), *t*(246.20) = 2.79, *p* = 0.006, 95% CI [0.09; 0.52], *d* = 0.03. However, this variable was not significantly correlated with brand attitude, *r*(256) = 0.06, *p* = 0.351, nor with any of the mediating variables (all *p* ≥ 0.113). Therefore, this variable was not included as a covariate in the subsequent main analyses.

### Hypotheses Testing

#### Conceptual Advertising Literacy

The results for *conceptual* advertising literacy activation are summarized in Table 3. With respect to H1a, exposure to the disclosure did not help children activate their conceptual advertising literacy. This was the case when conceptual advertising literacy activation was assessed via the indirect measurement task as well as when it was assessed with a self-reported questionnaire. Thus, H1a was not supported.

**TABLE 3:** Hypotheses testing for conceptual advertising literacy activation.

Dependent variable	Conceptual advertising literacy activation											
	Accuracy of categorization			Speed of categorization			Self-report			Brand attitude		
	*b (SE)*	*t*	*p*	*b (SE)*	*t*	*p*	*b (SE)*	*t*	*p*	*b (SE)*	*t*	*p*
Disclosure	−0.03 (0.52)	−0.05	0.957	−130.79 (461.81)	−0.28	0.777	−3.58 (2.20)	−1.63	0.104	−0.07 (0.12)	−0.58	0.561
Disclosure*dispositional	0.01 (0.10)	0.11	0.910	42.63 (86.88)	0.49	0.624	0.72 (0.41)	1.73	0.084			
Disclosure*dispositional*age	−0.03 (0.08)	−0.34	0.735	−25.77 (71.94)	−0.35	0.721	−0.49 (0.34)	−1.43	0.154			
**Advertising literacy activation**												
Accuracy of categorization										0.51 (0.27)	1.85	0.065
Speed of categorization										0.00 (0.00)	0.06	0.955
Self-report										0.07 (0.06)	1.21	0.227

**N* = 257. Model statistics for effects on accuracy of categorization, *F*(7,249) = 2.09, *p* = 0.046, *R*^2^ = 0.06. Model statistics for effects on speed of categorization, *F*(7,249) = 0.75, *p* = 0.633, *R*^2^ = 0.02. Model statistics for self-report, *F*(7,249) = 17.43, *p* < 0.001, *R*^2^ = 0.33. Model statistics for brand attitude, *F*(4,252) = 1.48, *p* = 0.208, R^2^ = 0.02. The analysis for conceptual advertising literacy activation was also conducted with the absolute scores for accuracy and speed of categorization of the advertising words (i.e., conceptual advertising literacy activation as assessed with the indirect measurement task). The results were the same.*

With respect to H2a, the relationship between exposure to the disclosure and conceptual advertising literacy activation was not moderated by children’s dispositional conceptual advertising literacy. This was the case when conceptual advertising literacy activation was assessed via the indirect measurement task as well as when it was assessed with a self-reported questionnaire (Table 3). This means that H2a was not supported.

With respect to H3a, there was no three-way interaction between exposure to the disclosure, children’s dispositional conceptual advertising literacy, and age-group on conceptual advertising literacy activation. This was the case when conceptual advertising literacy activation was assessed via the indirect measurement task as well as when it was assessed with a self-reported questionnaire (Table 3). This means that H3a was not supported.

Finally, with respect to H4a, children’s conceptual advertising literacy activation did not have a positive effect on brand attitude. This was the case when conceptual advertising literacy activation was assessed via the indirect measurement task as well as when it was assessed with a self-reported questionnaire, as can be seen in Table 3. Therefore, H4a was not supported.

#### Attitudinal Advertising Literacy

The results for *attitudinal* advertising literacy activation are summarized in Table 4. With respect to H1b, exposure to the disclosure did not help children activate their attitudinal advertising literacy. This was the case when attitudinal advertising literacy activation was assessed via an indirect measurement task as well as when it was assessed with a self-reported questionnaire. Thus, H1b was not supported.

**TABLE 4:** Hypotheses testing for attitudinal advertising literacy activation.

Dependent variable	Attitudinal advertising literacy activation											
	Accuracy of categorization			Speed of categorization			Self-report			Brand attitude		
	*b (SE)*	*t*	*p*	*b (SE)*	*t*	*p*	*b (SE)*	*t*	*p*	*b (SE)*	*t*	*p*
Disclosure	0.63 (1.47)	0.43	0.666	525.72 (1370.16)	−0.38	0.702	1.43 (1.97)	0.72	0.470	0.01 (0.12)	0.09	0.932
Disclosure*dispositional	−0.11 (0.34)	−0.33	0.742	174.05 (321.99)	0.54	0.589	−0.34 (0.46)	−0.74	0.459			
Disclosure*dispositional*age	0.05 (0.27)	0.17	0.866	−117.16 (249.10)	−0.47	0.639	0.33 (0.36)	0.94	0.348			
**Advertising literacy activation**												
Accuracy of categorization										0.05 (0.09)	0.55	0.580
Speed of categorization										0.00 (0.00)	−0.40	0.690
Self-report										−0.49 (0.07)	−7.41	<0.001

**N* = 210. Model statistics for effects on accuracy of categorization, *F*(7,202) = 2.38, *p* = 0.023, *R*^2^ = 0.08. Model statistics for effects on speed of categorization, *F*(7,202) = 0.68, *p* = 0.689, *R*^2^ = 0.02. Model statistics for self-report, *F*(7,202) = 3.47, *p* = 0.002, *R*^2^ = 0.11. Model statistics for brand attitude, *F*(4,205) = 13.86, *p* < 0.001, *R*^2^ = 0.21. Note that the number of participants in this analysis is lower, which is the result of calculating a difference score on the basis of categorization of positive words. Some children did not categorize a single positive word as being related to advertising. Therefore, their data is missing in this analysis. We also conducted the analysis with the absolute scores for accuracy and speed of categorization of the advertising words (i.e., attitudinal advertising literacy activation as assessed with the indirect measurement task), including all participants. The results were the same.*

With respect to H2b, the relation between exposure to the disclosure and attitudinal advertising literacy activation was not moderated by children’s dispositional attitudinal advertising literacy. This was the case when attitudinal advertising literacy activation was assessed via an indirect measurement task as well as when it was assessed with a self-reported questionnaire (Table 4). This means that H2b was not supported.

With respect to H3b, there was no three-way interaction between exposure to the disclosure, children’s dispositional attitudinal advertising literacy, and age-group on attitudinal advertising literacy activation. This was the case when attitudinal advertising literacy activation was assessed via an indirect measurement task as well as when it was assessed with a self-reported questionnaire (Table 4). This means that H3b was not supported.

Finally, with respect to H4b, children’s attitudinal advertising literacy activation had a negative effect on brand attitude, but only when it was assessed with a self-reported questionnaire (Table 4). This concurred with our expectation and implies that children who are more skeptical and more negative toward the presence of the brand in the video have a more negative brand attitude. The results for accuracy of categorizing the negatively valanced attitudinal words and speed of categorizing the negatively valenced attitudinal words were not significant. Therefore, H4b was only partially supported.

## Discussion

The first aim of this study was to investigate if a disclosure can stimulate children’s advertising literacy activation when they are exposed to in-vlog advertising. The second aim was to investigate if advertising literacy activation was related to children’s brand attitude. One of the main contributions of this study is that it assessed children’s advertising literacy activation with both an indirect measure [the Advertising Literacy Activation Task (ALAT)] (Hoek et al., 2019) and a direct measure (i.e., self-reported questionnaire). With regard to the first aim, the results showed that a disclosure prior to watching the vlog did not increase children’s advertising literacy activation. The results were unambiguous: the disclosure did not increase activation of conceptual advertising literacy nor of attitudinal advertising literacy. Both the indirect measurement task and the self-reported questionnaire showed no increase in activation. Thus, we conclude that the disclosure used here and in relation to this specific vlog was unsuccessful in activating children’s advertising literacy. This is an interesting finding because the manipulation in this study was successful, indicating that children in the disclosure condition noticed and remembered the disclosure.

A possible explanation for the fact that the disclosure did not activate children’s advertising literacy could be that the ‘hidden’ advertising in the vlog used here was actually not really hidden at all. For instance, brand recall and brand recognition were relatively high even in the condition without the disclosure (52.4% for brand recall and 88.1% for brand recognition). The high prominence of the brand could have triggered children to activate their advertising literacy. This is supported by the fact that advertising recognition and sponsorship recognition were equally high in both conditions (4.65 and 4.67, respectively, in the control condition, versus 4.88 and 4.76, respectively, in the experimental condition as measured on a scale from 1 to 6). Thus, it seems that children did not need the disclosure in order to recognize advertising and subsequently activate their advertising literacy.

It was expected that the relation between the disclosure and children’s advertising literacy activation was moderated by two variables: their dispositional advertising literacy and their age. There was no support for either moderator. Younger children (aged 7–11 years old) are considered cued processors (Roedder, 1981), meaning that they need a cue to activate information; however, the disclosure (i.e., cue) did not help these younger children in activating their advertising literacy. One explanation could be that the size of the two age groups was not the same (more younger than older children); this could limit the chance of finding a moderation effect of age. Another explanation could be that the highly prominent brand served as a cue for the younger participants that the vlog contained a commercial message (Friestad and Wright, 1994; Van Reijmersdal, 2009). This would have activated their advertising schema and made the disclosure unnecessary. This could explain why dispositional advertising literacy did not moderate the relation between the disclosure and advertising literacy activation either. If the commercial intent of the vlog was truly obvious due to the prominence of the brand, then it was easily recognizable even for children with less sophisticated levels of dispositional advertising literacy. This would result in equal levels of advertising literacy activation for children with lower and higher levels of dispositional advertising literacy. Another possible explanation for not finding a moderation effect for dispositional advertising literacy is that children’s scores on these variables, especially those on conceptual advertising literacy, were fairly high (mean = 5.13 on a scale ranging from 1 to 6), which might have led to a ceiling effect and as such, to a null effect of this variable.

Interestingly, there were no differences in the relationship between disclosure exposure and activation of advertising literacy for the two measurement methods used to assess activation (i.e., indirect measurement task and self-reported questionnaires). The use of the two different measurement methods produces the same results. Thus, we can conclude with more certainty that a disclosure, as used here, does not help children activate their advertising literacy when they are exposed to in-vlog advertising with highly prominent brand placement.

With regard to the second aim, our study showed that there was no positive relation between conceptual advertising literacy activation and brand attitude when activation was assessed with the indirect measurement task nor when it was assessed with a self-reported questionnaire. The reason for this could be that children’s brand attitude was already quite positive and thus a better realization that the vlog was made to make people like and buy the brand (i.e., higher conceptual advertising literacy activation) could not further increase this attitude.

For attitudinal advertising literacy we did find that stronger literacy activation led to a more negative attitude toward the brand, which is in line with previous studies (e.g., Rozendaal et al., 2016a; Vanwesenbeeck et al., 2016). However, we only found this effect when children’s attitudinal advertising literacy activation was assessed with a questionnaire and not with the indirect measurement task. This could indicate that a conscious evaluation that the presence of a brand in the vlog is dishonest and wrong may create a less favorable evaluation of the brand, whereas a subconscious evaluation of the brand in the context of the indirect task may not (Vandeberg, 2014). In other words, a subconscious evaluation of the brand may not be sufficiently strong to render a negative effect on brand attitude. This finding confirms the idea that indirect measurement tasks such as the ALAT may prevent *post hoc* rationalizations in participants about the persuasive intent of specific brand advertisements. Only when the child consciously judges the presence of the brand as something negative this may also have a negative effect on the evaluation of the brand.

Another explanation for the difference between the two measurement methods could be that there was a discrepancy between the level of measurement of advertising literacy activation (indirect) and the level of measurement of brand attitude (direct). An indirect way of measuring children’s advertising literacy activation may also need an indirect way of assessing their brand attitude, for instance with an Implicit Association Task (IAT; Nosek et al., 2007). This way, both variables are measured on the same, subconscious, level.

Finally, the difference between the two measurements methods could be explained by the wording and order of questioning that was used in the questionnaire. The questionnaire contained questions regarding the fairness of the presence of this specific brand, while the indirect measurement task assessed a more general skeptical attitude and disliking. Furthermore, the self-reported questions regarding activation of attitudinal advertising literacy were directly administered after the questions regarding brand attitude. It could be that children therefore linked these two concepts.

### Limitations and Future Research

Despite careful preparation, this study is subject to some limitations, leading to suggestions for future research. A first limitation is that the study implemented a single-message design. We decided to use a single-message design to keep the overall feasibility of the study high (i.e., chance of achieving required sample size) and the burden on the young participants acceptable. Moreover, by using a single-message design, we were better able to interpret any possible effects. However, a disadvantage of a single message design is that the effects, or lack of effects, could be driven by other message features (e.g., type of vlogger, gender of vlogger, narrative in the story). Future research could examine the hypothesized relationships for a variety of vlog messages. Doing so would allow to draw conclusions beyond one instance of a sponsored vlog.

Another limitation concerns the stimulus material, specifically the selected vlog and sponsoring brand, and the disclosure message used. In the current study, we used a vlog in which the brand was already quite prominent—this may have made the commercial intent of the vlog too obvious (Friestad and Wright, 1994; Van Reijmersdal, 2009). A suggestion for future research is to choose a vlog in which the brand and commercial intent is less prominent. If the commercial intent is less evident, then the effectiveness of the disclosure in increasing advertising literacy activation could be different because children actually need the disclosure to alert them to the commercial intent. This is also important because the commercial intent of in-vlog advertising is usually not as clearly presented as in the vlog used in the current study.

Another suggestion for future research related to the stimulus material is to work with a brand that is less familiar and less popular. In the current study, over 90% of the participants were familiar with the brand and the overall attitude toward the brand was very positive. This could be because children already had a favorable attitude toward the brand. As a consequence, it may have been difficult for the vlog and the disclosure to affect this existing attitude. The attitude toward a less familiar or unfamiliar brand may be easier to influence with exposure to a disclosure. Using a new and unfamiliar brand could therefore provide further insight into the relation between children’s dispositional advertising literacy, advertising literacy activation, advertising susceptibility, and the usefulness of a disclosure.

In future research, it is also important to focus on children’s understanding of the meaning of the disclosure. The present study used an explicit disclosure based on current regulations in the United States and Europe (e.g., FTC, 2013; WOMMA, 2013). The disclosure explicitly mentioned the name of the vlogger who created the video, the brand, the relationship between the two, and the fact that the brand paid for the advertising. However, it remains unknown if and how children understood the meaning of the disclosure. Differences in understanding of the disclosure may determine its effectiveness in activating persuasion knowledge. Future research could measure children’s understanding of the sponsorship disclosure used and include it as a control variable in the analyses. Furthermore, more research is needed into how disclosures should be designed and formulated to be understood by young viewers.

Another suggestion for future research with regard to the stimulus material used is to add a condition in which participants are shown a neutral ‘dummy’ message (e.g., “You are going to see an online video now”) at the start of the video. In doing so, a condition is added in which the structure of the message (i.e., a sentence presented on a black screen before the vlog content + vlog content) is exactly the same. As a result, several alternative explanations for a possible disclosure effect (e.g., the potential effect could be driven by the mere presence of a text before the video, not so much by the content of the text, and reading a text requires mental resources for processing which might consecutively affect children’s responses to the video) can be excluded.

With regard to the dependent measures for advertising literacy, future research could focus on other dimensions, representing more sophisticated levels of understanding (e.g., understanding of persuasive tactics used, understanding of the economic model of advertising) as well. In the current study, we chose to focus on two dimensions of conceptual ad literacy (i.e., advertising recognition and understanding of advertising’s selling and persuasive intent) and two dimensions of attitudinal advertising literacy (i.e., skepticism and critical attitude/disliking). The reason for this was that in the literature these variables are assumed to be important basic dimensions of advertising literacy, together forming the fundament of children’s advertising schemas (Rozendaal et al., 2011b; Hudders et al., 2017). Research shows that around the age of 7 (the minimum age of the children in the current study), most children have developed these fundamental elements of advertising literacy (De Jans et al., 2019b). For the current study, this was important because in order to activate advertising literacy from memory, children need to have a sufficient level of this literacy in place. The more sophisticated dimensions of advertising literacy, such as the understanding of persuasive tactics, develop at a significantly later age (Rozendaal et al., 2011a). Chances are that the youngest children in our sample had not yet developed this understanding, which means that they cannot activate it (regardless of whether they would actually do so if they were exposed to advertising).

Finally, there are some suggestions for future research with regard to the measurement methods used. In the current study two different measurement methods were used. Although it seems that the questionnaire measurement and indirect measurement yielded similar results, more research is needed on this topic. First, it is important to get a broader understanding whether the indirect and direct measure actually assess the exact same construct because this is not always clear (Fazio and Olson, 2003). It could be that the self-reported questionnaire measures a different level of advertising processing than the indirect measurement task. For instance, systematic processing may be better assessed with a direct measurement method while heuristic and automatic processing is better assessed with indirect measurement methods (Buijzen et al., 2010). Future research needs to test this.

Second, this work used a questionnaire measurement that provided support for the role of attitudinal advertising literacy activation in making children less susceptible to advertising effects whereas the indirect measurement did not. This finding could be further explored in future research that assesses brand attitude with both a questionnaire (as was done in this study) as well as with an indirect measurement task (e.g., an IAT or Approach-Avoidance Task). This will provide a better understanding of the relationship between different measurement methods in assessing children’s advertising literacy activation and their brand responses. For future research it is important to consider that the indirect measurement has several advantages over the questionnaire measurement—it is more difficult to give social desirable answers when an indirect measure is used (see Hoek et al., 2019 for an overview of advantages of an indirect measurement method as compared to a questionnaire measurement). However, indirect measurements also have their disadvantages. For instance, an indirect measurement task is more time-consuming and requires more technical skills from the researchers as compared to a questionnaire. Furthermore, it is paramount that the indirect measurement task is understood by the children as it is intended by the researchers.

### Implications

The results of this study have implications for the scientific community as well as government guidelines and advertisers. First, this study showed that a textual disclosure reading “[name vlogger] is being paid by [name company] to advertise in this video” does not necessarily increase the extent to which children activate their advertising literacy when exposed to in-vlog advertising. In this study, this may be due to the fact that the brand is very prominent in the video. Prominently placed brands seem to be an inherent trigger for children to activate their advertising literacy regardless of the presence of a disclosure. However, whether the prominence of the brand indeed made it easier for the children to activate their advertising literacy remains to be tested in future research. An important scientific implication of this study therefore is that the effectiveness of a disclosure for in-vlog advertising should always be considered in relation to the specific features of the brand placement and the vlog characteristics (e.g., prominence of the brand, familiarity of the brand and the vlogger, prior attitude to the brand and the vlogger). These features can make it either easier or harder for children to use their retrieval strategies in order to activate their advertising literacy schemas. From a theoretical perspective it is therefore important to take the characteristics of the message into account when studying children’s advertising information-processing and schema activation abilities.

Another theoretical implication is that this study showed that both direct and indirect measures are needed to get a comprehensive view of the relation between children’s advertising literacy and advertising susceptibility. The present study showed that direct and indirect measurements of advertising literacy activation reveal different processes through which children make sense of, and are affected by, advertising. That is, direct measures reveal more conscious and deliberate ways of advertising processing, while indirect measures reveal more subconscious and automatic ways of advertising processing. In daily life, children only use few mental resources to process advertising messages, meaning that children’s advertising processing is rather automatic (Buijzen et al., 2010; Rozendaal et al., 2011b). Therefore, direct measurement tools may not be capable of providing an answer to the question how children process advertising in a ‘natural’ state (e.g., when they are at home and not in an experimental setting, filling out a questionnaire). Indirect measures may be better capable to reveal this natural and more automatic way of advertising processing. Therefore, an important scientific implication is that to obtain full understanding of the role of advertising literacy in children’s susceptibility to advertising effects, both direct and indirect measures are needed.

Furthermore, our findings have important practical implications because such disclosures are typically required in current advertising guidelines in Europe and the United States. The current study showed that the disclosure, as used here, has failed to further raise the level at which children activate their advertising literacy while watching a vlog. This is not to say that all sponsorship disclosures are ineffective. It is important to first get an understanding under what circumstances sponsorship disclosures *are* effective in activating children’s advertising literacy. Based on these insights, government agencies might reconsider their guidelines and investigate whether other types of disclosures are more effective in increasing children’s advertising literacy activation, especially when the in-vlog advertising is relatively subtle and less prominent.

## Data Availability Statement

The raw data supporting the conclusions of this article will be made available by the authors, without undue reservation, to any qualified researcher.

## Ethics Statement

The studies involving human participants were reviewed and approved by Ethics Committee Social Science (ECSS) Radboud University Nijmegen. Written informed consent to participate in this study was provided by the participants’ legal guardian/next of kin.

## Author Contributions

RH, ER, HS, EvR, and MB designed the experiment and wrote the manuscript together. RH collected and analyzed the data.

## Conflict of Interest

The authors declare that the research was conducted in the absence of any commercial or financial relationships that could be construed as a potential conflict of interest.

## References

[B1] Amazeen M. A. Wojdynski B. W. (2018). The effects of disclosure format on native advertising recognition and audience perceptions of legacy and online news publishers. *Journalism* 1464884918754829 10.1177/1464884918754829

[B2] An S. Stern S. (2011). Mitigating the effects of advergames on children. *J. Adv.* 40 43–56. 10.2753/JOA0091-3367400103

[B3] Anderson R. C. (1978). “Schema-directed processes in language comprehension,” in *Cognitive Psychology and Instruction*, Vol. 5 eds Lesgold A. M. Pellegrino J. W. Fokkema S. D. Glaser R. (Boston, MA: Springer), 10.2307/1421580

[B4] Aronson E. Wilson T. D. Akert R. M. (2005). *Social Psychology*, 5th Edn New Jersey: Pearson Education International.

[B5] Atkinson R. C. Shiffrin R. M. (1968). Human memory: a proposed system and its control processes. *Psychol. Learn. Motivat.* 2 89–195. 10.1111/j.2007.0030-1299.15674.x

[B6] Banse R. Gawronski B. Rebetez C. Gutt H. Bruce Morton J. (2010). The development of spontaneous gender stereotyping in childhood: relations to stereotype knowledge and stereotype flexibility. *Dev. Sci.* 13 298–306. 10.1111/j.1467-7687.2009.00880.x 20136926

[B7] Baron A. S. Banaji M. R. (2006). The development of implicit attitudes: evidence of race evaluations from ages 6 and 10 and adulthood. *Psychol. Sci.* 17 53–58. 10.1111/j.1467-9280.2005.01664.x 16371144

[B8] Boerman S. C. Van Reijmersdal E. A. Neijens P. C. (2012). Sponsorship disclosure: effects of duration on persuasion knowledge and brand responses. *J. Commun.* 62 1047–1064. 10.1111/j.1460-2466.2012.01677.x

[B9] Boerman S. C. van Reijmersdal E. A. Neijens P. C. (2014). Effects of sponsorship disclosure timing on the processing of sponsored content: a study on the effectiveness of european disclosure regulations. *Psychol. Market.* 31 214–224.

[B10] Brace I. (2004). *Questionnaire design.* London: Kogan page.

[B11] Brehm J. W. Brehm S. S. (1981). *Psychological Reactance: A Theory Of Freedom And Control.* San Diego, CA: Academic Press.

[B12] Brown S. P. Stayman D. M. (1992). Antecedents and consequences of attitude toward the ad: a meta-analysis. *J. Consum. Res.* 19 34–51. 10.1086/209284

[B13] Buijzen M. Van Reijmersdal E. A. Owen L. H. (2010). Introducing the PCMC model: an investigative framework for young people’s processing of commercialized media content. *Commun. Theor.* 20 427–450. 10.1111/j.1468-2885.2010.01370.x

[B14] Campbell C. Evans N. J. (2018). The role of a companion banner and sponsorship transparency in recognizing and evaluating article-style native advertising. *J. Interact. Market.* 43 17–32. 10.1016/j.intmar.2018.02.002

[B15] Casey B. J. Tottenham N. Liston C. Durston S. (2005). Imaging the developing brain: what have we learned about cognitive development? *Trends Cognit. Sci.* 9 104–110. 10.1016/j.tics.2005.01.011 15737818

[B16] Childwise (2018). Available online at: http://www.childwise.co.uk/uploads/3/1/6/5/31656353/childwise_press_release_-_youtube_2018.pdf (accessed April 18, 2019).

[B17] Cvencek D. Meltzoff A. N. Greenwald A. G. (2011). Math-Gender stereotypes in elementary school children. *Child Dev.* 82 766–779. 10.1111/j.1467-8624.2010.01529.x 21410915

[B18] Dahlén M. Edenius M. (2007). When is Advertising Advertising? Comparing Responses to Non-Traditional and Traditional Advertising Media. *J. Curr. Issues Res. Adv.* 29 33–42. 10.1080/10641734.2007.10505206

[B19] D’Alessio M. Laghi F. Baiocco R. (2009). Attitudes toward TV advertising: a measure for children. *J. Appl. Dev. Psychol.* 30 409–418. 10.1016/j.appdev.2008.12.026 19492733

[B20] De Houwer J. (2006). “What are implicit measures and why are we using them?” In *Handbook Of Implicit Cognition And Addiction.* Eds Wiers R. W. Stacy A. W. (Thousand Oaks, CA: Sage Publications, Inc) 11–28.

[B21] De Jans S. Cauberghe V. Hudders L. (2019a). How an advertising disclosure alerts young adolescents to sponsored vlogs: the moderating role of a peer-based advertising literacy intervention through an informational vlog. *J. Adv.* 47 309–325. 10.1080/00913367.2018.1539363

[B22] De Jans S. Van de Sompel D. Hudders L. Cauberghe V. (2019b). Advertising targeting young children: an overview of 10 years of research (2006–2016). *Int. J. Adv.* 38 173–206.

[B23] De Jans S. Hudders L. Cauberghe V. (2017). Advertising literacy training: the immediate versus delayed effects on children’s responses to product placement. *Eur. J. Market.* 51 2156–2174. 10.1108/EJM-08-2016-0472

[B24] De Jans S. Vanwesenbeeck I. Cauberghe V. Hudders L. Rozendaal E. van Reijmersdal E. A. (2018). The development and testing of a child-inspired advertising disclosure to alert children to digital and embedded advertising. *J. Adv.* 47 255–269. 10.1080/00913367.2018.1463580

[B25] De Pauw P. Hudders L. Cauberghe V. (2017). Disclosing brand placement to young children. *Int. J. Adv.* 37 508–525. 10.1080/02650487.2017.1335040

[B26] Dunham Y. Baron A. S. Banaji M. R. (2006). From American city to Japanese village: a cross-cultural investigation of implicit race attitudes. *Child Dev.* 77 1268–1281. 10.1111/j.1467-8624.2006.00933.x 16999797

[B27] Evans N. J. Park D. (2015). Rethinking the Persuasion Knowledge Model: schematic antecedents and associative outcomes of persuasion knowledge activation for covert advertising. *J. Curr. Issues Res. Adv.* 36 157–176. 10.1080/10641734.2015.1023873

[B28] Fazio R. H. Olson M. A. (2003). Implicit measures in social cognition research: their Meaning and Use. *Annu. Rev. Psychol.* 54 297–327. 10.1146/annurev.psych.54.101601.145225 12172003

[B29] Fazio R. H. Sanbonmatsu D. M. Powell M. C. Kardes F. R. (1986). On the automatic activation of attitudes. *J. Pers. Soc. Psychol.* 50 229–238. 10.1037/0022-3514.50.2.229 3701576

[B30] Friestad M. Wright P. (1994). The Persuasion Knowledge Model: how people cope with persuasion attempts. *J. Consum. Res.* 21 1–31. 10.1086/209380

[B31] FTC (2013). *Com Disclosures. How to Make Effective Disclosure in Digital Advertising.* Available online at: http://www.ftc.gov/sites/default/files/attachments/press-releases/ftc-staff-revises-onlineadvertising-disclosure-guidelines/130312dotcomdisclosures.pdf (accessed May 08, 2019).

[B32] FTC Advertisement Endorsements (n.d.). Available online at: https://www.ftc.gov/news-events/media-resources/truth-advertising/advertisement-endorsements (accessed May 08, 2019).

[B33] Ham C. D. Nelson M. R. Das S. (2015). How to measure persuasion knowledge. *Int. J. Adv.* 34 17–53. 10.1080/02650487.2014.994730

[B34] Hayes A. F. (2017). *Introduction To Mediation, Moderation, And ConditionalProcess Analysis: A Regression-Based Approach (Second Edition).* New York: Guilford Publications.

[B35] Hoek R. W. Rozendaal E. van Schie H. T. Buijzen M. (2019). “The development and testing of the Advertising Literacy Activation Task,” in *Proceedings of the at the 69th Annual Meeting of the International Communication Association*, Washington, DC.

[B36] Hudders L. De Pauw P. Cauberghe V. Panic K. Zarouali B. Rozendaal E. (2017). Shedding new light on how advertising literacy can affect children’s processing of embedded advertising formats: a future research agenda. *J. Adv.* 46 333–349. 10.1080/00913367.2016.1269303

[B37] John D. R. (1999). Consumer Socialization of Children: a Retrospective Look At Twenty-Five Years of Research. *J. Consum. Res.* 26 183–213. 10.1086/209559

[B38] Kim S. J. Hancock J. T. (2016). How advertorials deactivate advertising schema: MTurk-based experiments to examine persuasion tactics and outcomes in health advertisements. *Commun. Res.* 44 1–27. 10.1177/0093650216644017

[B39] Leavitt K. Zhu L. Aquino K. (2016). Good without knowing it: subtle contextual cues can activate moral identity and reshape moral intuition. *J. Bus. Ethics* 137 785–800. 10.1007/s10551-015-2746-6

[B40] Lee J. E. Watkins B. (2016). YouTube vloggers’ influence on consumer luxury brand perceptions and intentions. *J. Bus. Res.* 69 5753–5760. 10.1016/j.jbusres.2016.04.171

[B41] Leys C. Ley C. Klein O. Bernard P. Licata L. (2013). Detecting outliers: do not use standard deviation around the mean, use absolute deviation around the median. *J. Exp. Soc. Psychol.* 49 764–766. 10.1016/j.jesp.2013.03.013

[B42] Machleit K. A. Wilson R. D. (1988). Emotional feelings and attitude toward the advertisement: the roles of brand familarity and repetition. *J. Adv.* 17 27–35. 10.1080/00913367.1988.10673121

[B43] Moses L. J. Baldwin D. A. (2005). What can the study of cognitive development reveal about children’s ability to appreciate and cope with advertising? *J. Pub. Polic Marke.* 24 186–201. 10.1509/jppm.2005.24.2.186

[B44] Nosek B. A. Greenwald A. G. Banaji M. R. (2007). The Implicit Association Test at Age 7: a Methodological and Conceptual Review. *Soc. Psychol. Unconsc. Automat. High. Ment. Process.* 265–292. 10.1016/j.mrfmmm.2009.01.007 19428370

[B45] OFCOM (2018). *Children And Parents: Media Use And Attitudes Report.* Available online at: https://www.ofcom.org.uk/__data/assets/pdf_file/0024/134907/children-and-parents-media-use-and-attitudes-2018.pdf (accessed April 19, 2019).

[B46] Ortells J. J. Kiefer M. Castillo A. Megías M. Morillas A. (2016). The semantic origin of unconscious priming: Behavioral and event-related potential evidence during category congruency priming from strongly and weakly related masked words. *Cognition* 146 143–157. 10.1016/j.cognition.2015.09.012 26412392

[B47] Pew Research Center. (2018). *Teens, Social Media & Technology 2018.* Available online at: http://www.pewinternet.org/2018/05/31/teens-social-media-technology-2018/ (2018, May)

[B48] Robinson M. D. Neighbors C. (2006). “Catching the mind in action: implicit methods in personality research and assessment,” in *Handbook of Multimethod Measurement in Psychology*, eds Eid M. Diener E. (Washington, DC: American Psychological Association), 115–125. 10.1037/11383-009

[B49] Roedder D. L. (1981). Age differences in children’s responses to television advertising: an information-processing approach. *J. Consum. Res.* 8 144–153. 10.1086/208850

[B50] Rozendaal E. Buijs L. Van Reijmersdal E. A. (2016a). Strengthening children’s advertising defenses?: the effects of forewarning of commercial and manipulative intent. *Front. Psychol.* 7:1186. 10.3389/fpsyg.2016.01186 27551271 PMC4976102

[B51] Rozendaal E. Opree S. J. Buijzen M. (2016b). Development and validation of a survey instrument to measure children’s advertising literacy. *Med. Psychol.* 19 1–29. 10.1080/15213269.2014.885843

[B52] Rozendaal E. Buijzen M. Valkenburg P. (2009). Do children’s cognitive advertising defenses reduce their desire for advertised products? *Communications* 34 287–303. 10.1515/COMM.2009.018

[B53] Rozendaal E. Buijzen M. Valkenburg P. (2011a). Children’s understanding of advertisers’ persuasive tactics. *Int. J. Adv.* 30 329–350.

[B54] Rozendaal E. Lapierre M. A. Van Reijmersdal E. A. Buijzen M. (2011b). Reconsidering advertising literacy as a defense against advertising effects. *Med. Psychol.* 14 333–354. 10.1080/15213269.2011.620540

[B55] Rozendaal E. Slot N. Van Reijmersdal E. A. Buijzen M. (2013). Children’s responses to advertising in social games. *J. Adv.* 42 142–154. 10.1080/00913367.2013.774588

[B56] Tessitore T. Geuens M. (2019). Arming consumers against product placement: a comparison of factual and evaluative educational interventions. *J. Bus. Res.* 95 38–48. 10.1016/j.jbusres.2018.09.016

[B57] Uytun M. C. (2018). “Development period of prefrontal cortex,” in *Prefrontal Cortex*, eds Starcevic A. Filipovic B. (Rijeka: IntechOpen), 10.5772/intechopen.78697

[B58] Van Berlo Z. M. C. Van Reijmersdal E. A. Rozendaal E. (2017). Weet wat er speelt: de rol van merkbekendheid in mobiele advergames. *Tijdschrift voor Communicatiewetenschap* 45 216–236.

[B59] Van Reijmersdal E. (2009). Brand placement prominence: good for memory! Bad for attitudes? *J. Advert. Res.* 49 151–153. 10.2501/s0021849909090199

[B60] Van Reijmersdal E. A. Boerman S. C. Buijzen M. Rozendaal E. (2017). This is advertising! Effects of disclosing television brand placement on adolescents. *J. Youth Adolesce.* 46 328–342. 10.1007/s10964-016-0493-3 27165259 PMC5241326

[B61] Van Reijmersdal E. A. Rozendaal E. Buijzen M. (2015). Boys’ responses to the integration of advertising and entertaining content. *Young Consum.* 16 251–263. 10.1108/YC-10-2014-00487

[B62] Van Reijmersdal E. A. Rozendaal E. Hudders L. Vanwesenbeeck I. Cauberghe V. Van Berlo Z. M. C. (2020). Effects of disclosing influencer marketing in videos: an eye tracking study among children in early adolescence. *J. Interact. Marke.* 49 94–106.

[B63] Vandeberg L. (2014). *Impliciet meten is weten?.* Amsterdam: Stichting Wetenschappelijk Onderzoek Commerciële Communicatie (SWOCC), 67.

[B64] Vandeberg L. Wennekers A. M. Murre J. M. J. Smit E. G. (2013). Implicit and explicit measures: what their dissociations reveal about the workings of advertising. *Adv. Adv. Res.* 4 73–85.

[B65] Vanwesenbeeck I. Ponnet K. Walrave M. (2017). Young adolescents’ advertising literacy and purchase intention in social network games: influence of perspective taking and need for cognition. *J. Consum. Behav.* 16 23–33. 10.1002/cb.1596

[B66] Vanwesenbeeck I. Walrave M. Ponnet K. (2016). Young adolescents and advertising on social network games: a structural equation model of perceived parental media mediation, advertising literacy, and behavioral intention. *J. Adv.* 45 183–197. 10.1080/00913367.2015.1123125

[B67] Verdoodt V. (2018). *Children’s Rights And Advertising Literacy In The Digital Era: Towards An Empowering Regulatory Framework For Commercial Communication.* Doctoral dissertation, Ghent University, Ghent.

[B68] Wojdynski B. W. Evans N. J. (2016). Going native: effects of disclosure position and language on the recognition and evaluation of online native advertising. *J. Adv.* 45 157–168.

[B69] WOMMA (2013). *Social Media Disclosure Guidelines.* Available online at: https://www.ana.net/miccontent/show/id/ii-womma-social-disclosure (accessed May 08, 2019).

[B70] Wright P. Friestad M. Boush D. M. (2005). The development of marketplace persuasion knowledge in children, adolescents, and young adults. *J. Pub. Polic. Mark.* 24 222–233. 10.1509/jppm.2005.24.2.222

[B71] Zeelenberg R. Pecher D. Shiffrin R. M. Raaijmakers J. G. W. (2003). Semantic context effects and priming in word association. *Psychon. Bull. Rev.* 10 653–660. 10.3758/BF03196528 14620360

